# Obtaining an Extract Rich in Phenolic Compounds from Olive Pomace by Pressurized Liquid Extraction

**DOI:** 10.3390/molecules24173108

**Published:** 2019-08-27

**Authors:** Inés Cea Pavez, Jesús Lozano-Sánchez, Isabel Borrás-Linares, Hugo Nuñez, Paz Robert, Antonio Segura-Carretero

**Affiliations:** 1 Departamento de Ciencia de los Alimentos y Tecnología Química, Facultad de Ciencias Químicas y Farmacéuticas, Universidad de Chile, Santos Dumont 964, Independencia, Santiago 8380494, Chile; 2 Center for Systems Biotechnology, Fraunhofer Chile Research, Av. Del Cóndor 844 floor 3, Santiago 8580704, Chile; 3 Department of Food Science and Nutrition, University of Granada, Campus Universitario s/n, 18071-Granada, Spain; 4 Research and Development of Functional Food Centre, Health Science Technological Park, Avda. del Conocimiento, n° 37, Ed. BioRegión, Armilla, Granada 18016, Spain; 5 Departamento de Agroindustria y Enología, Facultad de Ciencias Agronómicas, Universidad de Chile Casilla 1004 Santiago, Chile; 6 Department de Analytical Chemistry, Faculty of Sciences, University of Granada, Fuentenueva s/n, Granada 18071, Spain

**Keywords:** olive pomace, PLE, phenolic compounds, HPLC-DAD-ESI-TOF/MS

## Abstract

The olive oil industry produces large volumes of wastes, which are also potential sources of bioactive compounds by developing healthy and/or functional foods. Extraction of phenolic compounds from the residues of the olive oil is mainly carried out with solvents. However, there is currently a growing public awareness about the use of organic solvents in food processing, which has pointed out the need for the application of clean technologies such as pressurized liquid extraction (PLE). Therefore, the aim of this research was to optimize the phenolic compound extraction from olive pomace by PLE, establishing the qualitative and quantitative phenolic profile by HPLC-ESI-TOF/MS. The extraction design to recover phenolics from olive pomace demonstrates a great compositional variability of PLE extracts obtained under different experimental conditions. Indeed, quantitative results have pointed out the selectivity of PLE extraction when this technique is applied to the treatment of olive pomace. PLE-optimized conditions showed higher total phenolic compound content than conventional extraction (1659 mg/kg d.w. and 281.7 mg/kg d.w., respectively). Among these phenolics, the quantity of secoiridoids and flavonoids in the optimized PLE extract was three and four times higher than in conventional extracts. Furthermore, optimal PLE conditions allowed to obtain an enriched hydroxytyrosol extract which was not detected in the conventional one.

## 1. Introduction

The food industry produces large volumes of both solid and liquid wastes, which represent a disposal and potentially environmental pollution problem. However, they are also potential sources of bioactive compounds that can be recovered and used as valuable substances by developing healthy and/or functional foods [1,2]. In this sense, the production of extra virgin olive oil (EVOO) is associated with both the generation of large quantities of wastes and the loss of phenolic compounds during the process by the partitioning between oil and by-products (olive mill waste water, olive pomace, storage by-products, and filter cake) [3,4].

The olive pomace, or “Orujo”, is the solid by-product produced from the three-phase decanter process used in the olive oil industry. Olive pomace is composed of olive pulp, skin, stone and water. Concerning its chemical composition, high phenolic content has been reported by several authors, which reaches the level of 100 times higher than in EVOO [3,4].

Various studies have reported that olive oil consumption is associated with several health benefits, including the reduction of risk factors of coronary heart disease, the prevention of several chronic diseases (such as atherosclerosis), cancer, chronic inflammation, strokes, and other degenerative diseases. These beneficial health effects have been attributed, in part, to phenolic compounds [4,5,6].

Olive pomace phenolic compounds are a complex mixture of components that include hydroxytyrosol and tyrosol derivatives, iridoid precursors, secoiridoids and derivatives (oleuropein, oleuropein aglycone, ligstroside and its derivatives), phenylpropanoids (verbascoside and its derivatives), flavonoids (luteolin, apigenin, rutin, taxifolin and its derivatives), lignans (pinoresinol and derivatives), and phenolic acids (gallic acid, caffeic acid, cinnamic acid, *p*-coumaric acid, ferulic acid, vanillic acid, and shikimic acid) [7,8,9,10]. However, the concentration of these phenolic compounds has been reported to be affected by both agronomic and technological process conditions such as type of cultivar, ripening degree, different milling process, and edaphoclimatic factors [8,9].

For the extraction of olive phenolic compounds at laboratory-scale, different solvents have been used, such as methanol/water, ethyl acetate, propanol, acetone, or acetonitrile, but the effects of these compounds in humans and the environment are drawing attention. Thus, the industry has addressed its research to obtain bioactive compound-enriched extracts using different processes. Some reported methods for the extraction of phenolic compounds from olive oil wastes are: Solvent extraction [10,11,12,13,14], hydrothermal extraction [15,16], high pressure–high temperature reactor [17] extraction with subcritical water [18,19,20,21], microwave and ultrasound-assisted extraction [22,23], and absorbent resins or membrane separation [24,25,26]. Nevertheless, the industrial interest has been addressed to develop new processes based on more selective, environmentally-friendly, and cost-effective extraction techniques. Among these technologies, microwave-assisted extraction (MAE), supercritical fluid extraction (SFE) using CO_2_, and pressurized liquid extraction (PLE) have been applied to olive by-products [7,9].

PLE is considered as an advanced technology which uses liquid solvents at elevated temperature and pressure, improving the extraction performance as compared to those techniques carried out at room temperature and atmospheric pressure. The use of solvents at temperatures above their atmospheric boiling point improves the solubility and mass transfer properties [27]; moreover, it provides several advantages compared to the conventional technologies, showing better selectivity, extraction time reduction, and lower toxic organic solvent use [18,19,20,21]. Moreover, it is a technique effective not only as a laboratory tool, but also for agri-food industries [9].

Due to the above, the aim of this research was to: (a) Optimize the phenolic compound extraction from olive pomace by pressurized liquid extraction, (b) characterize the complete profile of phenolic compounds by HPLC-DAD-ESI-TOF/MS, and (c) quantify individual phenolics to determine an alternative methodology to recover bioactives from olive pomace.

## 2. Results and Discussion

### 2.1. Qualitative Characterization of Olive Pomace Phenolic Compounds Obtained by PLE

**Figure 1:**
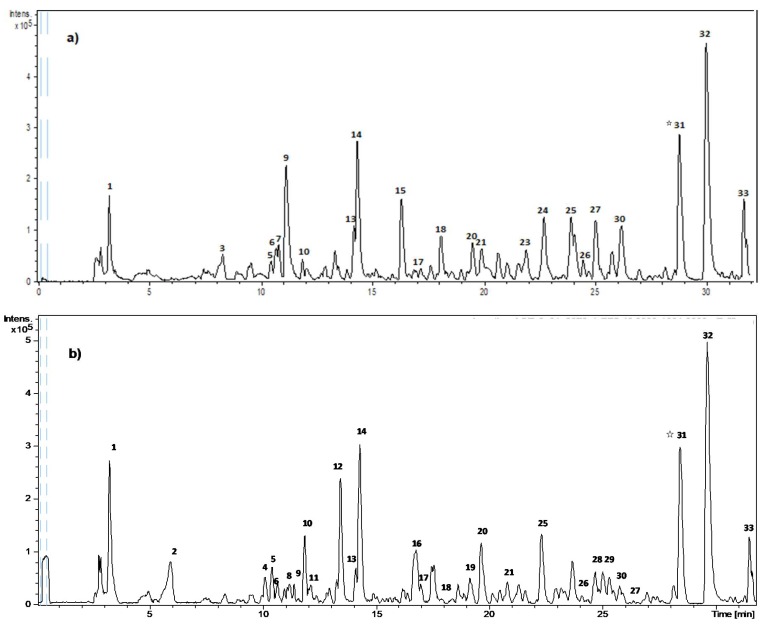
HPLC-DAD-ESI-TOF/MS chromatograms of olive pomace extracts. (**a**) Olive pomace extracts obtained by conventional extraction (OP), and (**b**) olive pomace extracts obtained by pressurized liquid extraction in optimal conditions (OP-PLE). **☆** Internal standard (Naringenin).

A central composite design for the extraction of olive pomace (OP) by PLE (OP-PLE) was carried out. Table 1 includes the tentative identification of phenolic compounds from olive pomace performed by HPLC-DAD-ESI-TOF/MS. Figure 1 shows the HPLC chromatogram. The phenolic compounds of OP were identified considering their retention times (Rt), UV-Vis spectrum, and MS spectrum. According to the chemical structure, phenolic compounds were classified in phenolic alcohols, secoiridoids, flavonoids, and lignans. Concerning phenolic alcohols, hydroxytyrosol and oxidized hydroxytyrosol were characterized in the analyzed samples. With regard to secoiridoids and derivatives, a total of seven compounds were identified: Oleoside, loganic acid, secoiridoid derived, D-OH-EA, hydroxy D-oleuropein aglycone, demethyl oleuropein, and an aldehydic form of decarboxymethyl elenolic acid. In addition, two flavonoids (luteolin and luteolin-7-glucoside) and two lignans (pinoresinol and acetoxypinoresinol) were also detected. A non-phenolic polar compound was also identified as quinic acid. Most of these compounds have been previously described in olive oil and olive by-products [4,8,19,23,27,28].

### 2.2. Quantitative Characterization of Olive Pomace Phenolic Compounds Obtained by PLE

**Figure 2:**
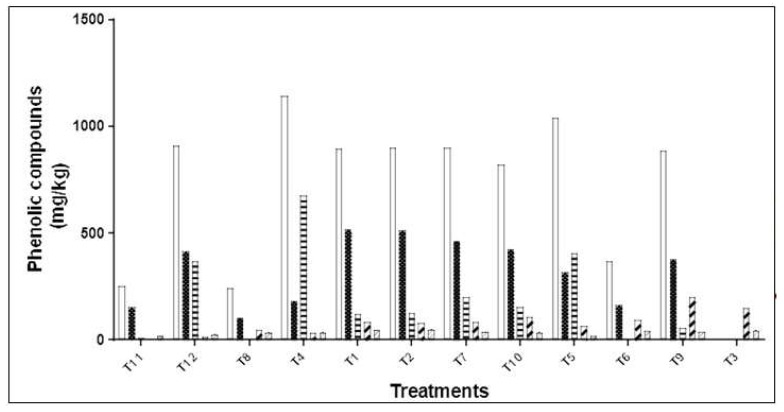
Phenolic compound content by groups, experimental design by olive pomace extracts obtained under different PLE extraction conditions. 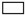 Total phenolic compounds; 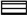 Phenolic alcohols; 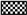 Secoiridoids; 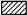 Flavonoids; and 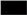 Lignans.

Table 2 shows the quantitation of individual phenolic compounds for each treatment of PLE system. The total phenolic content (PC) of OP-PLE extracts ranged from 241.1 to 1141.3 mg/kg d.w OP. Figure 2 includes the total phenolic compounds and the total content in secoiridoids, phenolic alcohols, flavonoids, and lignans. Secoiridoids reached the major concentration, ranging from 103.4 to 517 mg/kg d.w OP (42.9–57.6%, respectively). The highest amounts of secoiridoids were obtained for secoiridoid derivative (*m*/*z* 407), hydroxy oleuropein (*m*/*z* 555), and oleuropein (*m*/*z* 539). With regard to phenolic alcohols, hydroxytyrosol (*m*/*z* 153) and oxidized hydroxytyrosol (*m*/*z* 151) were identified and quantified. Their concentrations ranged from 0 to 675.6 mg/kg d.w OP. The highest phenolic alcohol contents were obtained in conditions T4, T5, and T12, where the higher temperature and water content, the higher the phenolic alcohol content. This result could be explained because the high extraction temperature and water content in solvent extraction could generate the hydrolysis of secoiridoids into phenolic alcohols and acidic moieties. These results agree with those reported by [29].

Flavonoids ranged from 3.1 to 199.9 mg/kg d.w OP (1.2–22.6%), luteolin being the flavonoid recovered in major amount in all the treatments. Concerning lignans, different amounts of pinoresinol and acetoxypinoresinol were quantitated in the extracts. Acetoxypinoresinol was the main phenolic, whose concentration ranged from 17.6 to 48.7 mg/kg d.w OP (1.7–6%).

The results showed that although the phenolic compound profile was similar for all treatments of the OP-PLE design, the phenolic compound content was different among runs. This behavior is characteristic of the PLE method, which presents differences in the selectivity of extraction [15]. The extraction factors such as temperature and water-ethanol ratio have been associated with the dielectric constant. Thus, the lower the dielectric constant, the higher the flavonoids content (r^2^ = 0.7336). However, no correlation was found between the dielectric constant and each phenolic group. It is important to consider that oxidized hydroxytyrosol (*m*/*z* 151), hydroxytyrosol (*m*/*z* 153), hydroxylated product of decarboxymethyl elenolic acid (*m*/*z* 199), hydroxy oleuropein (*m*/*z* 555), and luteolin-7-O-rutinoside (*m/z* 593) were identified in OP-PLE. These phenolic compounds have been reported to form by the oxidation reactions and/or hydrolysis of complex polyphenols [29].

### 2.3. Optimization of Recovering Phenolic Compounds from Olive Pomace by PLE Using Response Surface Methodology (RSM)

When characterizing the profile of phenolic compounds of the different treatments by PLE, the occurrence of hydroxytyrosol was observed in some extracts of olive pomace. Hydroxytyrosol presents comparative advantages over other polyphenols in olive residues, due to its higher antioxidant capacity and biological effects such as prevention of heart disease, tumor, and antithrombotic effect, among others [18,30]. In addition, the European Food Safety Agency (EFSA) approved health claims associated with consumption of hydroxytyrosol. In this context, the hydroxytyrosol content and its derivatives were used as response variables for the experimental design of olive pomace extracts by PLE.

Table 3 summarizes the experimental conditions (independent variables values: Ethanol content and extraction temperature) and response variables (hydroxytyrosol, hydroxy D-oleuropein aglycone, hydroxy-oleuropein, demethyloleuropein, decarboxymethyl oleuropein aglycone, and oleuropein content and yield). RSM was used to optimize each response variable considering linear, quadratic, and cross-product interactions of the independent variables at the 95% confidence level. The analysis of the variance (ANOVA) for the extraction of phenolic compounds from OP by PLE is shown in Table 4. Non-significant terms were removed from the equation, but when quadratic or cross-product interactions of the independent variables were significant, the linear forms of independent variables were considered in the quadratic equation, because they are fundamental elements of the mathematical model.

The hydroxytyrosol content ranged from 0 to 258.3 mg/kg (Table 3). The linear, quadratic, and cross-product forms of ethanol content (E) and extraction temperature (T) were significant on the hydroxytyrosol content. The model explained 78.8% of the variability in hydroxytyrosol content (R^2^ adj. d.f., Table 4).

The quadratic regression equation describing the effect of the independent variables on the hydroxytyrosol content was the following (Equation (1)):

(1) $$Hydroxytyrosol\;=\;-6.13428-1.65454\times T+2.0509\times E+0.0187878\times T^{2}+0.0178529\times T\times E-0.00503415\times E^{2}$$

**Figure 3:**
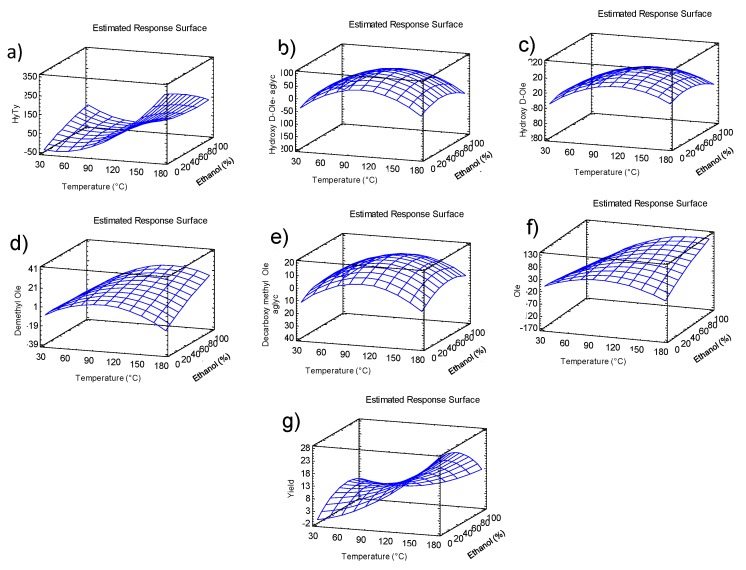
Response Surface graphs for (**a**) hydroxytyrosol, (**b**) hydroxy D-oleuropein aglycone, (**c**) hydroxy-oleuropein, (**d**) demethyl oleuropein, (**e**) decarboxymethyl oleuropein aglycone, (**f**) oleuropein content, and (**g**) yield.

As it can be seen in surface graph (Figure 3), the hydroxytyrosol content was higher when high extraction temperature and low ethanol content where applied. The hydroxy D-oleuropein aglycone and hydroxy D-oleuropein ranged from 0 to 95 mg/kg and from 0.4 to 134.6 mg/kg, respectively (Table 3). The effect of temperature and ethanol content quadratic forms were significant on hydroxy D-oleuropein aglycone content, while the quadratic form of temperature was only significant on hydroxy D-oleuropein content. In both response variables, the linear forms of temperature and ethanol content were considered in the quadratic equations. The model explained 75.1% and 64.7% of the variability in hydroxy D-oleuropein aglycone and hydroxy D-oleuropein content, respectively (R^2^ adj. d.f., Table 4). The quadratic regression equations describing the effect of the independent variables on hydroxy D-oleuropein aglycone (Equation (2)) and hydroxy D-oleuropein (Equation (3)) content are as follows:

(2) $$\mathrm{Hydroxy}\;D-\mathrm{oleuropein}\;\mathrm{aglycone}=-124.524\;+\;3.3054\times T\;+\;1.02943\times E\;–\;0.0155844\times T^{2}\;+\;0.00110948\times \;T\;\times E\;–\;0.0135149\times T^{2}$$

(3) $$\mathrm{Hydroxy}\;D-\mathrm{oleuropein}\;=\;-176.213\;+\;4.69159\times \;T\;+\;1.4106\times E\;–\;0.0203739\times {T\;}^{2}+\;0.000338833\times \;T\;\times E\;–\;0.0188999\times E^{2}$$

**Figure 4:**
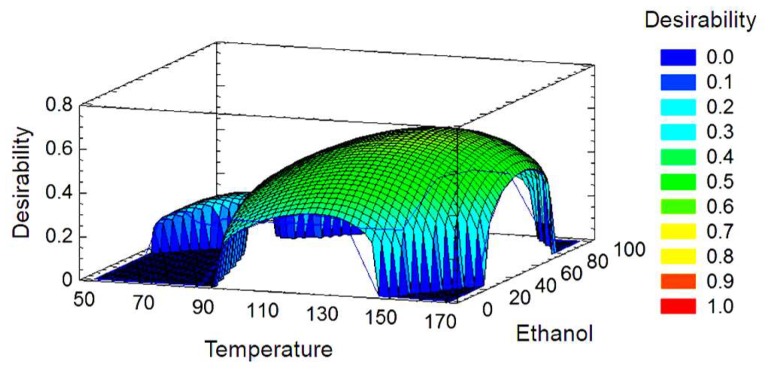
Desirability function overlay surfaces plots of olive pomace extracts by pressurized liquid extraction.

The response surface graphs showed that the highest hydroxy D-oleuropein aglycone and hydroxy D-oleuropein content was achieved with intermediate values of temperature and ethanol content (Figure 4).

The demethyl oleuropein concentration ranged from 0 to 21.6 mg/kg (Table 3). The quadratic form of temperature was significant; however, the linear form was considered in the quadratic equations. The linear form of ethanol content and cross-product forms of ethanol as extracting solvent and temperature were significant for demethyl oleuropein content. The model explained 80.6% of the variability (R2 adj. for d.f., Table 4).

The quadratic regression equation of demethyl oleuropein content (Equation (4)) is the following:

(4) $$\mathrm{Demethyl}\;\mathrm{oleuropein}=\;-24.3166\;+\;0.642713\times \;T\;–\;0.028972\times E\;–\;0.00327204\times {\;T}^{2}\;+\;0.0023143\times \;T\;\times E\;–\;0.000741822\times E^{2}$$

As can be seen in the response surface graph (Figure 3), the highest demethyl oleuropein content was achieved with high temperature and high ethanol content.

The decarboxymethyl oleuropein aglycone concentration ranged from 0 to 19.1 mg/kg (Table 3). Only the quadratic form of temperature and ethanol content were significant; however, the linear form and cross-product forms of ethanol as extracting solvent and temperature were considered in the quadratic equations for decarboxymethyl oleuropein aglycone. The model explained 83.6% of the variability (R2 adj. for d.f., Table 4).

The quadratic regression equation of decarboxymethyl oleuropein aglycone content (Equation (5)) is:

(5) $$\mathrm{Decarboxymethyl}\;\mathrm{oleuropein}\;\mathrm{aglycone}\;=\;-28.6763\;+\;0.667961\times T\;+\;0.272767\times E\;–\;0.00318761\times {\;T\;}^{2}+\;0.00059887\times T\;\times E\;–\;0.00299909\times E^{2}$$

As can be seen in the response surface graph (Figure 3), the highest decarboxymethyl oleuropein aglycone content was achieved with intermediate values of temperature and ethanol content.

The oleuropein concentration ranged from 3.7 to 113.4 mg/kg (Table 3). The linear, quadratic form of temperature, linear form of ethanol content and cross-product forms of ethanol as extracting solvent and temperature were significant, so were considered in the quadratic equations for oleuropein content. The model explained 88.6% of the variability (R2 adj. for d.f., Table 4).

The quadratic regression equation of oleuropein content is the following (Equation (6)):

(6) $$\mathrm{Oleuropein}\;=\;-47,8575\;+\;1,86207\times \;T\;-\;0,487302\times E\;-\;0,00932032\times {\;T}^{2}\;+\;0,0120913\times \;T\;\times E\;-\;0,00409461\times E^{2}$$

As can be seen in the response surface graph (Figure 3), the highest oleuropein content was achieved with intermediate temperature and high ethanol content.

According to the information described above, it is observed that the highest hydroxytyrosol content was obtained in the treatments with high extraction temperatures (T4 and T5), while hydroxy-D-oleuropein aglycone, dimethyl oleuropein, and decarboxymethyl-oleuropein aglycone were not detected in these experiments. In contrast, hydroxytyrosol was not detected in the treatments carried out at the lowest temperatures (T6, T8, and T11). These results show that high extraction temperatures would cause rupture of secoiridoides to phenolic alcohols [14].

Regarding oleuropein, the highest value was obtained in the treatment performed at high temperature with high ethanol content (T9).

In relation to yield, this ranged from 3.7 to 25.7% (Table 4). The linear, quadratic, and cross-product forms of ethanol as extracting solvent and temperature were significant for yield (Equation (7)). The model explained 93.6% of the variability (R2 adj. for d.f.) in yield (Table 4).

(7) $$\mathrm{YIELD}\left[\;\%\;\right]=\;-1,57556\;+\;0,00941454\;\times \;T+\;0,278964\times E+\;0,000838349\times T^{2}\;-\;0,0013601\times T\;\times E\;-\;0,00176315\times E^{2}$$

As can be seen in the surface graph (Figure 3), the yield showed the highest values when using high temperature extraction and a minor proportion of ethanol in the extraction solvent (T4 and T5).

Finally, multiple optimization taking into account all response variables was evaluated (Desirability Function). Figure 4 shows the surface response graphic. The optimal conditions for the extraction of phenolic compounds and yield were 136.5 °C and 52.3% of ethanol in the ethanol:water mixture. In the graph of the response surface, the optimum values are located at the yellow zone with 0.7 of desirability.

### 2.4. Characterization of Olive Pomace Extracts Obtained Under Optimal PLE Conditions (OP-PLE)

Figure 1 shows the HPLC-DAD-ESI-TOF/MS chromatograms and the tentative identification of phenolic compounds in the olive pomace extract obtained by conventional extraction system (OP) and olive pomace extract by optimal PLE (OP-PLE). In both olive pomace extracts, it is possible to identify different phenolic compounds which have previously been reported in olive fruits and olive derivatives [31].

Table 5 shows phenolic compound content of both OP and OP-PLE extracts. The total phenolic compound content in OP-PLE extract was higher than in OP (1659 mg/kg d.w. and 281.7 mg/kg d.w., respectively), showing a total phenolic compound value equivalent to 588.9% with respect to the extract obtained by conventional extraction. This value is much higher than those previously reported (201–256%) [8]. These results can be attributed to the high temperature and pressure of the PLE extraction method, process conditions that improve the interaction capacity between the phenolic compounds and the extraction solvent (ethanol and water mixture). On the other hand, the surface tension and the viscosity of the extraction solvent are reduced by the increase of temperature, which leads to better wetting and penetration in the matrix (olive pomace), thus increasing the mass transfer and therefore the extraction of phenolic compounds [17,19,22,23,27,28].

Higher hydroxytyrosol contents than that of this study were reported in extracts of olive pomace (var. Picual) (2800 mg hydroxytyrosol/kg d.w.) using ethanol–water as solvent and static-dynamic process [7,18]. However, the initial phenolic compounds concentration in a matrix depends on several agronomic (including cultivar, ripening stage, geographic origins, and tree irrigations, among others) and technological factors (like the process temperature and water content) [3,8,32]. Future studies are warranted to compare both techniques using the same matrix.

In OP extract, the main phenolic compound class was secoiridoids, with more than half of the total phenolic content (54.8%): Secoiridoid derivated 1 (16.9%), derived from oleuropein aglycone (8.6%), oleuropein (7.7%), and ligstroside (7.0%). These results are in agreement with other studies, in which the main phenolic compounds are secoiridoids (between 50 and 70%) [8]. Flavonoids are the second chemical group in importance (23%), with luteolin being the main compound of this subclass (17.7%). In contrast, in OP-PLE extract obtained under optimum conditions, the main phenolic compound class is phenolic alcohols with 42.5% (phenolic compound class that were not found in the OP), followed by secoiridoids (26.4%) and flavonoids (16.4%), with luteolin being the main phenolic compound (13.2%). The appearance of phenolic alcohols and the increase of the elenolic acid derivatives in OP-PLE could be related to degradation reactions of secoiridoids, followed by several reactions, such as oxidation, hydration, and loss of the carboxylic and carboxymethyl groups due to the high temperatures used in the extraction method [33,34,35].

On the other hand, the OP-PLE extract obtained under optimal conditions had a processing yield of 17.2%, which is higher to that found in olive extracts obtained with the PLE method.

## 3. Materials and Methods

### 3.1. Samples

Olive pomace (Orujo, var. arbequina) waste from a three-phase decanter was provided by Olivos Ruta del Sol Company (33°31’48.0´´S 71°40´ 52.6´´W, Santa Cruz, Bernardo O´Higgins Region, Chile) (May, 2018). Olive pomace (OP) was dried in a freeze-dryer (IlShinBioBase Co. Ltd. Modelo FD5508, Dongduchun City Kyunggi-do, Korea) and stored in bags hermetically sealed at –20 °C in dark conditions until the extract preparation.

### 3.2. Chemicals

Hexane, ethanol, methanol, and sodium hydroxide were purchased from Panreac (Barcelona, Spain). Acetic acid was acquired from Fluka (Steinheim, Germany). Double-deionized water with conductivity lower than 18.2 MV was obtained with a Milli-Q system (Millipore, Bedford, MA, USA). Standards of hydroxytyrosol, caffeic acid, luteolin, apigenin, quinic acid, and naringenin were purchased from Sigma-Aldrich (St. Louis, MO, USA), and (+)-pinoresinol was acquired from Arbo Nova (Turku, Finland). Oleuropein and luteolin-7-O-glucoside were purchased from Extrasynthese (Lyon, France).

### 3.3. Conventional Extraction Procedure of Phenolic and Other Polar Compounds from Olive Pomace (OP)

Conventional extraction of phenolic compounds from olive pomace was performed by solid–liquid extraction. Lyophilized olive pomace (5.0 g d.w.) was soaked for 120 min with 20 mL of a mixture of methanol:water (80:20). After this, the samples were centrifuged at 10,000 rpm for 15 min, and the supernatants were collected and filtered through a 0.45 μm filter. Each procedure was carried out in triplicate. The OP extracts were frozen at –20 °C until analysis.

### 3.4. Extraction of Phenolic Compounds from Olive Pomace by PLE (OP-PLE)

PLE was performed using a Dionex ASE 350 Accelerated Solvent Extractor (Thermo Fisher Scientific, Leicestershire, UK). All extractions were done using 34-mL extraction cells, containing 5 g of lyophilized olive pomace mixed homogeneously with 10 g of sand. Prior to extraction of phenolic compounds, a preliminary clean-step based on the use of n-hexane as the solvent and 1500 psi at room temperature as the experimental conditions was carried out to remove the lipophilic fraction from the olive pomace. After this step, the extraction of phenolic compounds from the olive pomace was performed according to a central composite design, with a total of 12 runs (4 experimental points, 4 axial points, and 4 central points). The ethanol percentage (0 to 100%) and temperature (40 to 176 °C) were evaluated as independent variables to cover a wide range of dielectric constants (from 19 to 65.5 F/m, Table 6). The pressure and extraction time were 1500 psi and 20 min, respectively. All of the experiments were conducted randomly to avoid systematic errors.

The obtained extracts were protected from light, filtered through a 0.45 µm regenerated cellulose filter and evaporated under vacuum in Speed Vac (Thermo Scientific, Leicestershire, UK). Yield (Y), hydroxytyrosol, and hydroxytyrosol-containing compounds were used as response variables for the experimental design of olive pomace extracts by PLE (hydroxityrosol (HYTY), hydroxyoleouropein aglycone, hydroxyoleuropein, demethyl oleuropein, decarboxymethyl oleuropein, and oleouropein).

Response surface methodology (RSM) was applied to determine the optimal conditions for the OP-PLE system by multiple response optimization using the desirability function (DF) where the response variables were maximized. The data were fitted to a second-order regression model according to Equation (8).

(8) $$Y=b_{0}+\overset{2}{\underset{i=1}{\sum }}b_{i}X_{i}+\overset{2}{\underset{i=1}{\sum }}b_{ii}X_{i}^{2}+\overset{1}{\underset{i=1}{\sum }}\overset{2}{\underset{j=i+1}{\sum }}b_{ij}X_{i}X_{j}$$

where Y is the response; subscripts i and j range from 1 to the number of variables (*n* = 2); $b_{0}$ is the intercept term; $b_{i}$ values are the linear coefficients; $b_{ij}$ values are the quadratic coefficients; and $X_{i}$ and $X_{j}$ are the levels of independent variables.

### 3.5. High-Performance Liquid Chromatography Coupled to Diode Array Detection and Electrospray Time-of-Flight Mass Spectrometry (HPLC-DAD-ESI-TOF/MS)

The HPLC analyses were performed in a high-performance resolution liquid chromatography (HPLC) system (Agilent Technologies, Waldbronn, Germany) equipped with a vacuum degasser, autosampler, a binary pump, and diode-array-detector (DAD). This equipment was coupled to a time-of-flight mass spectrometry (TOF-MS) microTOF (Bruker Daltonik, Bremen, Germany). The TOF mass spectrometer was equipped with a model G1607A ESI interface (Agilent Technologies, Palo Alto, CA, USA) operating in negative ion mode. The analytical column used was a 150 mm × 4.6 mm internal diameter, 1.8-µm Zorbax Eclipse Plus C18 (Agilent Technologies, Palo Alto, CA, USA).

The flow rate was 0.5 mL/min, and the temperature of the column was maintained at 25 °C. The mobile phase was water with 0.25% acetic acid (Solvent A) and methanol (Solvent B) eluted according to the following multistep gradient: 0 min, 5% Solvent B; 7 min, 35% Solvent B; 13 min, 45% Solvent B; 18.5 min, 50% Solvent B; 22 min, 60% Solvent B; 29 min, 95% Solvent B; 36 min, 5% Solvent B; and the injection volume was 10 µL.

The compounds separated were monitored with DAD (240 and 280 nm) and MS. At this stage, the use of a splitter was required for the coupling with the MS detector, as the flow arriving to the TOF detector had to be 0.25 mL/min in order to ensure reproducible results and a stable spray. External mass spectrometer calibration was performed with sodium acetate clusters (5 mM sodium hydroxide in water/2-propanol 1/1 (*v*/*v*), with 0.2% of acetic acid) in high-precision calibration (HPC) regression mode.

The phenolic compound quantification was performed with calibration curves elaborated with commercial standards: Hydroxytyrosol was used to quantify hydroxytyrosol and oxidized hydroxytyrosol; tyrosol was used to quantify tyrosol and ligstroside; caffeic acid was used to quantify verbascoside; oleuropein was used to quantify oleuropein, its isomers and derivatives, oleosides and elenolic acid derivatives, and secoiridoids; pinoresinol was used to quantify pinoresinol and acetoxypinoresinol; luteolin was used to quantify luteolin; and luteolin 7-glucoside was used to quantify luteolin 7-glucoside and luteolin 7-rutinoside. All phenolic compound standard solutions were prepared at a concentration of 1000 mg/L by dissolving the appropriate amount of the compound in methanol and then serially diluting to working concentrations (0.5 to 50 mg/L). Naringenin was added at 25 µg/mL and used as internal standard.

### 3.6. Statistical Analysis

Linear regression (95% confidence limit) was used to determine the correlation between dielectric constant and each phenolic compound class for the design from olive pomace extracts by PLE. A one-way analysis of variance (ANOVA) and Duncan’s multiple range test were performed to test for differences in phenolic compound content between olive pomace extracts by pressurized liquid extraction in optimal conditions (OP-PLE) and olive pomace extract by conventional extraction (OP). The statistical analyses were performed using Statgraphics Centurion XV (StatPoint Inc., Warrenton, VA, USA, 2011).

## 4. Conclusions

The extraction design of phenolic compounds from olive pomace by PLE demonstrates the great variability of extracts that can be obtained by changing the extraction conditions, presenting differences in the selectivity of extraction. The olive pomace extract obtained under optimized conditions showed a higher concentration in phenolics than that obtained by conventional extraction, besides presenting hydroxytyrosol, a compound which was not found in the extraction by maceration. The olive pomace extract obtained under optimized PLE conditions can be used in the design of bioactive food ingredients, as well as nutraceuticals.

## Figures and Tables

**Table 1:** Tentative identification of phenolic compounds and their derivatives in olive pomace by HPLC-DAD-ESI-TOF/MS.

Peak	Tentative Identification	Molecular Formula	Rt (min)	*m/z*
1	Quinic acid	C_7_H_12_O_6_	3.3	191.0561
2	Oxidized hydroxytyrosol	C_8_H_8_O_3_	6.0	151.0401
3	Unknown 1	C_16_H_26_O_11_	8.3	393.1428
4	Vanillic acid	C_8_H_8_O_4_	10.2	167.0350
5	Oleoside/secologanoside or isomer 1	C_16_H_22_O_11_	10.5	389.1114
6	Loganic acid	C_16_H_24_O_10_	10.7	375.1318
7	Unknown 2	C_15_H_26_O_9_	10.8	349.1526
8	Hydroxytyrosol	C_8_H_10_O_3_	11.0	153.0557
9	Secoiridoid derived	C_17_H_28_O_11_	11.2	407.1604
10	Decarboxylated form of hydroxyl elenolic acid	C_10_H_14_O_5_	11.9	213.0768
11	Hydroxylated product of decarboxymethyl elenolic acid	C_9_H_12_O_5_	12.1	199.0618
12	Unknown 3	C_8_H_8_O_3_	13.5	151.0401
13	Oleoside/secologanoside or isomer 2	C_16_H_22_O_11_	14.1	389.1089
14	Unknown 4	C_9_H_12_O_4_	14.3	183.0663
15	Unknown 5	C_16_H_26_O_10_	16.3	377.1493
16	Hydroxy oleuropein	C_25_H_32_O_14_	17.0	555.1719
17	Demethyl oleuropein	C_24_H_30_O_13_	17.5	525.1614
18	Aldehydic form of decarboxymethyl elenolic acid	C_10_H_16_O_5_	18.2	215.0925
19	Luteolin-7-O-rutinoside	C_27_H_30_O_15_	19.5	593.1510
20	Unknown 6	C_36_H_42_O_14_	19.6	685.2469
21	Luteolin-7-glucoside	C_21_H_20_O_11_	20.1	447.0933
22	Unknown 7	C_38_H_26_O_8_	21.1	609.1555
23	Unknown 8	C_38_H_26_O_6_	21.9	577.1657
24	Oleuropein	C_25_H_32_O_13_	22.9	539.1770
25	Luteolin-7-glucoside or isomer	C_21_H_20_O_11_	20.1	447.0933
26	Pinoresinol	C_20_H_22_O_6_	24.0	357.1344
27	Acetoxypinoresinol	C_22_H_24_O_8_	24.5	415.1390
28	Unknown 9	C_31_H_36_O_11_	25.3	583.2123
29	Unknown 10	C_31_H_36_O_12_	25.6	583.2123
30	Ligstroside	C_25_H_32_O_12_	26.3	523.1821
31	Naringenin (Internal standard)	C_15_H_12_O_5_	28.9	271.0893
32	Luteolin	C_15_H_10_O_6_	30.1	285.0405
33	Apigenin	C_15_H_10_O_5_	31.7	269.0451

**Table 2:** Phenolic compound content in experimental design by olive pomace extracts obtained under different PLE extraction conditions.

Tentative Identification	T_1_	T_2_	T_3_	T_4_	T_5_	T_6_	T_7_	T_8_	T_9_	T_10_	T_11_	T_12_
	108 °C 50% EtOH	108 °C 50% EtOH	108 °C 100% EtOH	164 °C 10% EtOH	176 °C 50% EtOH	51 °C 90% EtOH	108 °C 50% EtOH	40 °C 50% EtOH	164 °C 90% EtOH	108 °C 50% EtOH	51 °C 10% EtOH	108 °C 0% EtOH
	(mg/kg d.w.)											
**Quinic acid**	**127.5 ± 6.9**	**137.2 ± 3.4**	**145.8 ± 0.9**	**217.8 ± 11.5**	**231.6 ± 1.3**	**71.4 ± 1.1**	**118.7 ± 0.9**	**56.3 ± 1.4**	**215.5 ± 10.9**	**107.3 ± 0.0**	**68.4 ± 0.8**	**89.2 ± 2.8**
Oxidized hydroxytyrosol	94 ± 9	100 ± 7	-	458 ± 27	148.4 ± 0.5	-	176 ± 4	-	-	131.3 ± 0.0	10.3 ± 0.7	363 ± 12
Oleoside/secologanoside or isomer 1	61 ± 1	60 ± 2	44.5 ± 0.6	12.6 ± 0.4	32.1 ± 0.5	28.6 ± 0.9	61.7 ± 0.8	10.2 ± 0.1	77.9 ± 0.6	49.1 ± 0.0	32 ± 1	44 ± 2
Loganic acid	-	-	8.3 ± 0.3	9.0 ± 0.4	6.9 ± 0.2	11.1 ± 0.2	-	8.4 ± 0.2	6 ± 1	-	12.8 ± 0.4	8.6 ± 0.6
Hydroxytyrosol	28 ± 2	24 ± 1	51 ± 2	218 ± 1	258 ± 5	-	23 ± 1	-	55 ± 3	22.4 ± 0.0	-	6.9 ± 0.0
Secoiridoid derived	98.9 ± 0.5	95 ± 1	67 ± 1	119 ± 3	143 ± 4	74.2 ± 0.3	100 ± 2	62.0 ± 0.2	84 ± 3	97 ± 4	83.3 ± 0.2	73 ± 2
D-OH-EA	2.9 ± 0.2	2.6 ± 0.1	1.7 ± 0.0	-	-	6.8 ± 0.5	2.7 ± 0.2	-	-	0.6 ± 0.0	1.7 ± 0.2	-
Hydroxy D-oleuropein aglycone	70 ± 1	70 ± 2	12.1 ± 0.2	-	-	3.8 ± 0.1	73 ± 2	1.1 ± 0.0	9.9 ± 0.5	61 ± 2	3.3 ± 0.4	66 ± 3
Hydroxy oleuropein	123 ± 2	125 ± 4	22.3 ± 0.7	25.5 ± 0.9	62 ± 3	2.3 ± 0.3	105 ± 2	0.4 ± 0.0	33.6 ± 0.3	101.6 ± 0.1	6.6 ± 0.5	133 ± 1
Demethyl oleuropein	19.7 ± 0.6	19.5 ± 0.8	21.6 ± 0.9	-	-	-	15.3 ± 0.5	-	20.9 ± 0.5	10.9 ± 0.1	-	5.0 ± 0.5
Aldehydic form of decarboxymethyl elenolic acid	9.4 ± 0.7	9.5 ± 0.8	3.2 ± 0.2	-	-	-	-	5.1 ± 0.1	-	7.6 ± 0.0	8.8 ± 0.1	3.0 ± 0.1
Luteolin-7-glucoside	19.2 ± 0.6	10.0 ± 0.9	21.0 ± 0.3	-	-	2.6 ± 0.3	8.3 ± 0.3	-	35 ± 1	37.5 ± 0.0	-	-
Decarboxymethyl oleuropein aglycon	19.1 ± 0.8	18.1 ± 0.7	10 ± 1	-	-	0.5 ± 0.0	13.9 ± 0.6	-	4.9 ± 0.1	12 ± 3	-	4.8 ± 0.3
Oleuropein	80.0 ± 0.3	81 ± 5	79 ± 1	15 ± 1	63.2 ± 0.9	15.2 ± 0.3	67.5 ± 0.7	6.0 ± 0.3	113 ± 3	60.0 ± 0.3	3.7 ± 0.2	56 ± 2
Pinoresinol	11.4 ± 0.5	10.9 ± 0.3	10.7 ± 0.8	3.7 ± 0.3	-	7.8 ± 0.2	8.3 ± 0.4	4.6 ± 0.0	10.9 ± 0.0	6.9 ± 0.0	-	1.4 ± 0.0
Acetoxypinoresinol	33.6 ± 0.5	37.8 ± 0.8	30.1 ± 0.5	28.9 ± 0.2	17.6 ± 0.2	33.7 ± 0.8	28.0 ± 0.4	29.3 ± 0.4	27.5 ± 0.3	25 ± 1	17.8 ± 0.4	23 ± 1
Ligstroside	32 ± 1	31 ± 1	29.4 ± 0.5	-	8.5 ± 0.4	19.8 ± 0.4	24.4 ± 0.1	10.2 ± 0.3	24.0 ± 0.4	22.1 ± 0.3	-	19.4 ± 0.5
Luteolin	66 ± 1	68 ± 2	127.5 ± 0.8	34 ± 1	67.4 ± 0.7	71 ± 1	74.0 ± 0.5	41.0 ± 0.6	158 ± 1	67.9 ± 0.0	3.1 ± 0.0	12.7 ± 0.1
Apigenin	-	-	-	-	-	17.4 ± 0.2	-	6.5 ± 0.2	6.9 ± 0.3	0.5 ± 0.0	-	-
Secoiridoids (mg/kg)	517 ± 1	511 ± 10	299 ± 2	182 ± 5	315.7 ± 0.5	162 ± 3	463.1 ± 0.4	103 ± 1	375 ± 3	422.5 ± 0.1	153 ± 1	413 ± 6
(%)	57.6 ± 0.2	56.8 ± 0.9	43.7 ± 0.4	15.9 ± 0.4	30.4 ± 0.0	44.3 ± 0.7	51.5 ± 0.0	42.9 ± 0.5	42.4 ± 0.4	51.4 ± 0.0	60.5 ± 0.5	45.4 ± 0.7
Phenolic alcohols (mg/kg)	122 ± 4	124 ± 1	51.1 ± 0.5	676 ± 11	407 ± 7	-	199 ± 2	-	55 ± 3	153.7 ± 0.0	10.3 ± 0.8	370 ± 3
(%)	13.6 ± 0.5	13.8 ± 0.6	7.5 ± 0.1	59 ± 1	39.1 ± 0.6	-	22.2 ± 0.2	-	6.3 ± 0.4	18.7 ± 0.0	4.1 ± 0.3	40.7 ± 0.3
Flavonoids (mg/kg)	85.7 ± 0.5	78 ± 2	148 ± 2	34 ± 1	67.4 ± 0.7	91.3 ± 0.1	82.3 ± 0.9	47.5 ± 0.3	199.9 ± 0.0	105.9 ± 0.0	3.1 ± 0.0	12.7 ± 0.1
(%)	9.6 ± 0.1	8.7 ± 0.1	21.7 ± 0.3	3.0 ± 0.1	6.5 ± 0.1	24.9 ± 0.0	9.1 ± 0.1	19.7 ± 0.1	22.6 ± 0.0	12.9 ± 0.0	1.2 ± 0.0	1.4 ± 0.0
Lignans (mg/kg)	45 ± 2	49 ± 1	41 ± 1	33 ± 2	17.6 ± 0.2	41.5 ± 0.9	36.3 ± 0.8	33.9 ± 0.4	38.4 ± 0.1	32 ± 1	17.8 ± 0.3	25 ± 1
(%)	5.0 ± 0.2	5.4 ± 0.1	6.0 ± 0.2	2.9 ± 0.2	1.7 ± 0.0	11.3 ± 0.2	4.0 ± 0.1	14.1 ± 0.2	4.3 ± 0.0	3.9 ± 0.2	7.1 ± 0.1	2.7 ± 0.1
Total phenolic compounds (mg/kg)	897 ± 3	890 ± 12	685 ± 9	1141 ± 10	1039 ± 11	366 ± 2	900 ± 18	241 ± 7	885 ± 9	821.0 ± 0.1	252 ± 3	910 ± 2
(%)	100	100	100	100	100	100	100	100	100	100	100	100

EtOH: Ethanol; d.w.: Dry weight.

**Table 3:** Phenolic compound content and yield of experimental design.

Treatments			HyTy	Hydroxy D-Ole aglyc	Hydroxy- Ole	Demethyl Ole	Decarboxy methyl Ole aglyc	Oleuropein	Yield
	Temperature (°C)	Ethanol (%)	(mg/kg d.w.)						(%)
T1	108(0)	50(0)	28 ± 2	70 ± 1	123 ± 2	19.7 ± 0.6	19.1 ± 0.8	80.0 ± 0.3	10.2
T2	108(0)	50(0)	24 ± 1	70 ± 2	124 ± 4	19.5 ± 0.8	18.1 ± 0.7	81 ± 5	13.3
T3	108(0)	100(α)	51 ± 2	12.1 ± 0.2	22.3 ± 0.7	21.6 ± 0.9	10 ± 1	79 ± 1	5.5
T4	164(1)	10(-1)	218 ± 11	-	25.5 ± 0.9	-	-	15 ± 1	21.7
T5	176(α)	50(0)	258 ± 5	-	62 ± 3	-	-	63.2 ± 0.9	25.7
T6	51(-1)	90(1)	-	3.8 ± 0.1	2.3 ± 0.3	-	0.5 ± 0.0	15.2 ± 0.3	3.7
T7	108(0)	50(0)	23 ± 1	73 ± 2	105 ± 2	15.3 ± 0.5	13.9 ± 0.6	67.5 ± 0.7	10.7
T8	39(-α)	50(0)	-	1.1 ± 0.0	0.4 ± 0.0	-	-	6.0 ± 0.3	5.9
T9	164(1)	90(1)	55 ± 3	9.9 ± 0.5	33.6 ± 0.3	20.9 ± 0.5	4.9 ± 0.1	113 ± 3	11.8
T10	108(0)	50(0)	22.4 ± 0.0	61 ± 2	101.6 ± 0.1	10.9 ± 0.1	12 ± 3	60.0 ± 0.3	11.0
T11	51(-1)	10(-1)	-	3.3 ± 0.4	6.6 ± 0.5	-	-	3.7 ± 0.2	6.1
T12	108(0)	0(-α)	6.9 ± 0.0	66 ± 3	133 ± 1	5.0 ± 0.5	4.8 ± 0.3	56 ± 2	9.5

T: Treatments; d.w.: Dry weight; HyTy: Hydroxytyrosol; Hydroxy D-Oleuropein aglycone: Hydroxy D-oleuropein aglycone; Hydroxy-Ole: Hydroxy-oleuropein; Demethylole: Demethyloleuropein; decaboxymethyl Ole aglyc: Decarboxymethyl oleuropein aglycone.

**Table 4:** Analysis of variance (ANOVA) for the olive pomace extracts obtained by PLE.

Source	Sum of Squares	d.f.	Mean Square	F-Ratio	*p*-Value	R^2^	R^2^ adj. d.f.
**Hydroxytyrosol**							
A: Temperature	42324.8	1	42324.8	42324.85	0.0000∗	88.473	78.8671
B: Ethanol	1623.56	1	1623.56	1623.56	0.0000∗		
AA	14278.4	1	14278.4	14278.42	0.0000∗		
AB	6511.91	1	6511.91	6511.91	0.0000∗		
BB	409.411	1	409.411	409.41	0.0000∗		
Lack-of-fit	9738.63	3	3246.21	3246.21	0.0000*		
Pure error	94.9681	3	31.656				
Total (corr.)	82912.1	11					
**Hydroxy D-oleuropein aglycone**							
A: Temperature	118.618	1	118.618	0.36	0.5704	86.4431	75.1457
B: Ethanol	523.819	1	523.819	1.59	0.2541		
AA	10127.7	1	10127.7	30.75	0.0015∗		
AB	25.1495	1	25.1495	0.08	0.7916		
BB	1619.86	1	1619.86	4.92	0.0479*		
Lack-of-fit	1016.74	3	338.913	1.64	0.3481		
Pure error	1976.45	6	329.408				
Total (corr.)	14578.9	11					
**Hydroxy-oleuropein**							
A: Temperature	3656.18	1	3656.18	3,.6	0.1065	80.7716	64.748
B: Ethanol	2401.14	1	2401.14	2.37	0.175		
AA	17567.8	1	17567.8	17.31	0.0059∗		
AB	2.34565	1	2.34565	0	0.9632		
BB	3117.69	1	3117.69	3.07	0.1302		
Lack-of-fit	4635.83	3	1545.28	6.46	0.0798		
Pure error	6090.85	6	1015.14				
Total (corr.)	31676.3	11					
**Demethyl oleuropein**							
A: Temperature	69.2627	1	69.2627	3.98	0.0929	89.4383	80.6369
B: Ethanol	239.089	1	239.089	13.76	0.0100∗		
AA	461.585	1	461.585	26.56	0.0021∗		
AB	109.428	1	109.428	6.3	0.0460∗		
BB	6.55101	1	6.55101	0.38	0.5618		
Lack-of-fit	52.3368	3	17.4456	1.01	0.4976		
Pure error	104.287	6	17.3811				
Total (corr.)	987.409	11					
**Decarboxymethyl oleuropein aglycone**							
A: Temperature	14.449	1	14.449	1.61	0.251	91.0723	83.6326
B: Ethanol	13.6533	1	13.6533	1.52	0.263		
AA	425.539	1	425.539	47.53	0.0005∗		
AB	7.32752	1	7.32752	0.82	0.4005		
BB	81.121	1	81.121	9.06	0.0237∗		
Lack-of-fit	7.96575	3	2.65525	0.25	0.8548		
Pure error	53.7187	6	8.95311				
Total (corr.)	601.709	11					
**Oleuropein**							
A: Temperature	5650.06	1	5650.06	38.15	0.0008∗	93.7987	88.631
B: Ethanol	1826.83	1	1826.83	12.34	0.0126∗		
AA	3976.57	1	3976.57	26.85	0.0021∗		
AB	2987.01	1	2987.01	20.17	0.0041∗		
BB	76.7892	1	76.7892	0.52	0.4985		
Lack-of-fit	329.614	3	109.871	0.59	0.6639		
Pure error	888.52	6	148.087				
Total (corr.)	14328	11					
**Yield**							
A: Temperature	295.563	1	295.563	108.09	0.0000∗	96.5258	93.6305
B: Ethanol	23.3825	1	23.3825	8.55	0.0265∗		
AA	27.7676	1	27.7676	10.15	0.0189∗		
AB	37.7949	1	37.7949	13.82	0.0099∗		
BB	36.3875	1	36.3875	13.31	0.0107∗		
Lack-of-fit	10.3376	3	3.44588	1.83	0.3165		
Pure error	16.4071	6	2.73452				
Total (corr.)	472.249	11					

d.f.: degrees of freedom. ∗ Statistically significant.

**Table 5:** Phenolic compound content of olive pomace extracts by pressurized liquid extraction in optimal conditions (OP-PLE) and olive pomace extract by conventional extraction (OP).

Compound Names	OP (d.w.)		OP-PLE (d.w.)	
	(mg/kg)	(%)	(mg/kg)	(%)
Quinic acid	40.2 ± 0.2 ^a^	14.1 ± 0.9 ^a^	223 ± 20 ^b^	13.6 ± 0.4 ^a^
Oxidized hydroxytyrosol	- ^a^	- ^a^	638 ± 16 ^b^	38.5 ± 0.2 ^b^
Oleoside/secologanoside or isomer 1	4.8 ± 0.3 ^a^	1.7 ± 0.1 ^a^	33.9 ± 0.2 ^b^	2.1 ± 0.0 ^b^
Hydroxytyrosol	- ^a^	- ^a^	67 ± 2 ^b^	4.0 ± 0.1 ^b^
Secoiridoid derived	47.6 ± 2.0 ^b^	16.9 ± 0.9 ^b^	9.7 ± 0.4 ^a^	0.6 ± 0.0 ^a^
Decarboxylated form of hydroxyl elenolic acid	2.9 ± 0.4 ^a^	1.1 ± 0.1 ^a^	60 ± 1 ^b^	3.6 ± 0.1 ^b^
Hydroxylated product of decarboxymethyl elenolic acid	- ^a^	- ^a^	17.2 ± 0.3 ^b^	1.1 ± 0.1 ^b^
Demethyl oleuropein	2.9 ± 0.4 ^b^	0.8 ± 0.1 ^b^	UDL ^a^	UDL ^a^
Elenolic acid glucoside or isomer 1	0.7 ± 0.2 ^a^	0.3 ± 0.1 ^a^	33.4 ± 0.5 ^b^	2.0 ± 0.1 ^b^
Oleoside/secologanoside or isomer 2	15 ± 2 ^a^	5.7 ± 0.5 ^b^	45.5 ± 0.4 ^b^	2.8 ± 0.1 ^a^
Hydroxy oleuropein	- ^a^	- ^a^	100 ± 1 ^b^	5.9 ± 0.2 ^b^
Aldehydic form of decarboxymethyl elenolic acid	13 ± 1 ^b^	4.7 ± 0.3 ^b^	UDL ^a^	UDL ^a^
Luteolin-7-O-rutinoside	- ^a^	- ^a^	2.3 ± 0.1 ^b^	0.1 ± 0.0 ^b^
Luteolin-7-O-glucoside	2.5 ± 0.2 ^a^	0.9 ± 0.0 ^a^	21 ± 2 ^b^	1.3 ± 0.1 ^b^
Oleuropein	22 ± 3 ^a^	7.7 ± 0.9 ^b^	94 ± 1 ^b^	5.6 ± 0.0 ^a^
Ligstroside	20 ± 2 ^a^	7.0 ± 0.6 ^b^	16.5 ± 0.8 ^a^	1.0 ± 0.1 ^a^
Pinoresinol	4.7 ± 0.3 ^b^	1.7 ± 0.1 ^b^	2.9 ± 0.3 ^a^	0.2 ± 0.0 ^a^
Acetoxypinoresinol	18 ± 1 ^b^	6.5 ± 0.3 ^a^	14.8 ± 0.4 ^a^	0.9 ± 0.0 ^a^
Luteolin	50 ± 1 ^a^	17.7 ± 0.4	221 ± 4 ^b^	13.2 ± 0.2
Apigenin	12.4 ± 0.5 ^a^	4.4 ± 0.2	29.8 ± 0.1 ^b^	1.9 ± 0.1
Total phenolic compounds	282 ± 12 ^a^	100 ^a^	1659 ± 31 ^b^	100 ^a^
Secoiridoids	154 ± 9 ^a^	55 ± 1 ^b^	435 ± 10 ^b^	26.4 ± 0.3 ^a^
Phenolic alcohols	- ^a^	- ^a^	702 ± 14 ^b^	42.5 ± 0.2 ^b^
Flavonoids	65 ± 2 ^a^	23.0 ± 0.5 ^b^	271 ± 7 ^b^	16.4 ± 0.1 ^a^
Lignans	23 ± 2 ^b^	8.1 ± 0.3 ^b^	17.6 ± 0.6 ^a^	1.1 ± 0.0 ^a^

OP: Olive pomace; PLE: Pressurized liquid extraction; d.w.: Dry weight; UDL: Under detection level. All the variables were tested in three independent cultures for each experiment. Values are means ± SD. Different letters represent level of significance: *p* < 0.05.

**Table 6:** Central composite Design 2^2^ with star points from PLE.

Treatments	Temperature (°C)	Ethanol (%)	Dielectric Constant (F/m)
T_1_	108	50	36.1
T_2_	108	50	36.1
T_3_	108	100	19.0
T_4_	164	10	39.2
T_5_	176	50	28.9
T_6_	51	90	28.6
T_7_	108	50	36.1
T_8_	40	50	46.0
T_9_	164	90	20.8
T_10_	108	50	36.1
T_11_	51	10	65.6
T_12_	108	0	53.5
